# Circ-STC2 promotes the ferroptosis of nucleus pulposus cells via targeting miR-486-3p/TFR2 axis

**DOI:** 10.1186/s13018-023-04010-1

**Published:** 2023-07-21

**Authors:** Liangping Xiong, Xiaoyan Li, Xi Hua, Zhonglai Qian

**Affiliations:** 1 Department of Orthopedic Surgery, The First Affiliated Hospital of Soochow University, 899 Pinghai Road, Suzhou, 215006 Jiangsu China; 2 Department of Orthopedic Surgery, The First People’s Hospital of Jiande, Hangzhou, China; 3 Department of Orthopedic Surgery, Affiliated Hospital of Jining Medical University, Jining, China

**Keywords:** Intervertebral disc degeneration, Circ-STC2, Ferroptosis, TFR2, Nucleus pulposus cell

## Abstract

**Background:**

Low back pain (LBP) has become the second leading cause of disability worldwide, which has brought great economic burden to people. It is generally believed that intervertebral disc degeneration (IDD) is the main cause of LBP. This study aimed to explore the role of circ-STC2 in the pathogenesis of IDD.

**Methods:**

Nucleus pulposus cells (NPCs) were treated with T-Butyl Hydrogen Peroxide (TBHP) to establish IDD model in vitro. RT-qPCR was performed to detect mRNA expressions. The cell viability was detected with CCK-8 assay. The levels of lactate dehydrogenase (LDH), malondialdehyde (MDA), Fe^2+^ and glutathione (GSH) of NPCs were measured by corresponding kits. The protein expressions were determined by western blot. Dual-luciferase reporter and RNA pull-down assays were conducted to verify the relationship between circ-STC2 or transferrin recepto 2 (TFR2) and miR-486-3p.

**Results:**

Circ-STC2 and TFR2 expressions were up-regulated in IDD tissues, and miR-486-3p expression was down-regulated. Knockdown of circ-STC2 promoted the cell viability and inhibited the ferroptosis of the NPCs. The GSH levels, and glutathione peroxidase 4 (GPX4) and solute carrier family 7 member 11 (SLC7A11) protein expressions were increased, the LDH, MDA and Fe^2+^ levels and achaete-scute complexlike 4 (ASCL4) protein expressions were decreased after circ-STC2 knockdown. Knockdown of miR-486-3p abrogated the si-circ-STC2 effects and overexpression of TFR2 reversed the miR-486-3p mimic effects.

**Conclusions:**

Circ-STC2 inhibits the cell viability, induced the ferroptosis of the TBHP treated NPCs via targeting miR-486-3p/TFR2 axis.

**Supplementary Information:**

The online version contains supplementary material available at 10.1186/s13018-023-04010-1.

## Introduction

Low back pain (LBP) has become the second leading cause of disability worldwide, which has brought great economic burden to people [1]. It is generally believed that intervertebral disc degeneration (IDD) is the main cause of LBP [2]. The intervertebral disc is the largest non vascular tissue in the human body, consisting of a central nucleus pulposus, a peripheral fibrous ring, and an upper and lower cartilage endplate. The occurrence of IDD is induced by various factors, such as the unique anatomical structure and nutrient supply of intervertebral disc, inflammatory response, death of nucleus pulposus cells (NPCs), synthesis and catabolism of extracellular matrix (ECM)[3, 4].

Cell death is one of the important links in the occurrence and development of IDD [5]. In recent years, more and more researchers have found that in addition to apoptosis, there are some regulatory forms of cell death that induce cell death, such as ferroptosis [6]. In 2012, Dixon et al. [7] proposed the concept of ferroptosis for the first time. In recent years, it has been found that inhibiting ferroptosis can significantly reduce the ischemic injury of liver, kidney, brain and heart in mouse models, indicating that ferroptosis plays a key role in the ischemic injury diseases of these organs [8, 9]. Although the ferroptosis has been confirmed to be related to the pathogenesis of renal and brain injury disease [10–12], the participation of ferroptosis in the spinal diseases, including IDD, remains unclear.

Circular RNA (circRNA) is a kind of non-coding RNA, which exists in a large number of natural organisms and is charactered by richness, conservatism and structural stability [13, 14]. In 2013, Hansen et al. [15] first reported that circRNA contains a large number of miRNA response elements, which can be used as miRNA sponge to regulate target genes by playing the role of competitive endogenous RNA (ceRNA) at the post transcriptional level. It has been widely studied and confirmed that circRNAs play an important role in the occurrence and development of cardiovascular diseases, diabetes and cancer [16–18]. In 2018, Cheng et al. [19] first reported that circRNA functions as ceRNA to regulate NPCs apoptosis and participate in IDD. In addition, Chang et al. [20] confirmed that circ-STC2 functions as a key circRNA to participate in regulation of IDD progression via bioinformatics analysis. However, the specific mechanism of circ-STC2 in the IDD development is still unclear. Therefore, this study aimed to explore the roles of circ-STC2 in IDD and the underlying mechanisms.

## Materials and methods

### Tissues samples

The patients were recruited from The First Affiliated Hospital of Soochow University. We collected 50 NP tissues from IDD patients and 50 NP tissues of normal intervertebral disc from the patients who underwent idiopathic scoliosis surgery. All patients agreed to the performance of this study and signed the consent form. What’s more, this study was approved by the ethics committee of The First Affiliated Hospital of Soochow University. The clinical characteristics of all participants were showed in Table 1.

**Table 1:** Clinical characteristics

Parameters	Normal (*n* = 50)	IDD (*n* = 50)	*P* value
Age (years)			0.4233
≤ 45	28	24	
> 45	22	26	
Sex			0.2298
Female	21	27	
Male	29	23	
Body mass index			0.6853
≤ 24 kg/m^2^	20	22	
> 24 kg/m^2^	30	28	
Degeneration level			–
L3/4	–	18	
L4/5	–	20	
L5/S1	–	12	
MRI grade			–
G (I/II)	–	27	
G (IV/V)	–	23	
Sampling location	First Affiliated Hospital of Soochow University		

### Cell culture and treatment

The normal NP tissue was cut into small pieces and transferred into a sterile centrifuge tube. Trypsin (0.05%, 1 mL) was added to digest, then the digestion was terminated with DMEM/F-12 complete culture medium. Next, the tube was centrifuged to remove the supernatant (1500 r/min, 5 min). The cells were digested with trypsin for 6 h. Then the cells were washed and cultured in DMEM/F-12 complete medium containing 10% FBS and 1% penicillin streptomycin (37 ℃, 5% CO_2_). After 3 days, the floating tissue was removed. The culture medium was replaced every 3 days to observe the cell adhesion and growth. Then the cells were treated with 100 μM T-Butyl Hydrogen Peroxide (TBHP; Sigma) for 4 h to establish the IDD model in vitro. In addition, the TBHP-treated NPCs were treated with 10 μM simvastatin (SIM; 1002347-74-1, MedChemExpress), 5 μM ferrostatin-1 (Fer-1; HY‐100579, MedChemExpress), 20 μg/ml iron-saturated holo-transferrin (HTF; T1283, Sigma) and 4 μM Erastin (571203-78-6, MedChemExpress) for 24 h, respectively.

### Cell transfection

After TBHP treatment, The NPCs were seeded into 96-well plates at a density of 6 × 10^4^ cells/well, when cultures reached 40–60% density, the NPCs were transfected with si-cric-STC2, miR-486-3p mimic and inhibitor, and TFR2 overexpressed vector and their negative controls (si-nc, mimic nc, and inhibitor nc), respectively (GenePharma, Shanghai, China). These plasmids were transfected into NPCs with Lipofectamine 2000 reagent (Invitrogen, California, USA) according to the manufacturer instructions. After 8 h of transfection, culture media were replaced with fresh media. When confluent, transfected NPCs were passaged for further experiments.

### CCK-8 method

A CCK-8 kit (APExBIO, USA) was used to detect cell viability. NPCs were cultured in a 96-well plates for 48 h after treatment. Then CCK-8 reagents were added to each well and incubated for 2 h. Finally, the absorbance (A) value were detected at 450 nm of the multifunctional enzyme labeling instrument.

### RT-qPCR

The total RNA of the IDD samples and NPCs was extracted by using Trizol (Boyetime, Nantong, China). Then the concentration and purity the RNA were detected by UV spectrophotometer. The RNA was reverse transcribed by using the instructions of the reverse transcription kit (PrimeScript RT Master Mix (Perfect Real Time) RR036A, Takara, Dalian, China). Then the real-time fluorescence quantitative PCR reaction was performed with SYBR® Premix Ex Taq™ quantitative kit (Takara, Dalian, China). The reaction conditions were: 95 ℃, 30 s; 95 ℃, 5 s; 60 ℃, 30 s, 40 cycles. GAPDH was selected as Housekeeping gene. The relative expressions was calculated with 2^−ΔΔCt^ method. The Primer sequences were as follows (5'- > 3'):

Circ-STC2, Forward Primer GGGTGTGGCGTGTTTGAATG; Reverse Primer TTTCCAGCGTTGTGCAGAAAA.miR-486-3p, Forward Primer GGCAGCTCAGTACAGGATAAA; Reverse Primer CGGGGCAGCUCAGUACAGGAU.

TFR2, Forward Primer GGTGACCAATGCTCAGGACTT; Reverse Primer CAGGTGTGTAGGGGTCTCCA.

GAPDH, Forward Primer TGTGGGCATCAATGGATTTGG; Reverse Primer ACACCATGTATTCCGGGTCAAT.

U6, Forward Primer CTCGCTTCGGCAGCACA; Reverse Primer AACGCTTCACGAATTTGCGT.

### Determination of Fe, LDH, MDA and GSH levels

The levels of Fe^2+^ (MAK025), lactate dehydrogenase (LDH, 11,644,793,001), malondialdehyde (MDA, MAK085) and glutathione (GSH, MAK440) in NPCs were measured according to the manufacturer's instructions using commercial kits (Sigma-Aldrich, USA).

### TdT-mediated biotinylated nick end-labeling (TUNEL) assay

Cell death of NPCs was determined by In Situ Cell Death Detection Kit (Roche, Switzerland). Briefly, cells were trypsinized and then fixed with 4% paraformaldehy. After that, the cells were depermeated with 0.1% Triton X-100 (Beyotime Biotech, China), and incubated with H_2_O_2_. After washing with PBS, cells were incubated with TUNEL reaction mixture at 37 °C for 1 h. Then cells were incubated with terminal dexynucleotidyl transferase (TdT) reaction cocktail at 37 °C for 1.5 h. After washed with PBS thrice, cells were stained with Hematoxylin for 3 min. Finally, cells were captured using a fluorescent microscope.

### Western blot assay

RIPA (Sigma, USA) reagents were used to extract protein from NPCs. Protein concentration was determined by a BCA kit (Sigma). Additionally, proteins (40 µg/per lane) were isolated by 15% SDS-PAGE gel. After that, the proteins were transferred onto PVDF membranes (Bio-rad, USA). The membranes were blocked with 5% skimmed milk for 2 h and incubated with primary antibodies including SLC7A11 (1:600, Abcam, USA, ab275411), GPX4 (1: 800, Abcam, USA, ab262509), ACSL4 (1: 1000, Abcam, USA, ab155282), and GAPDH (1: 1500, Abcam, USA, ab8245) at 4 °C overnight, followed by incubation with secondary goat anti-mouse antibody to immunoglobulin G (IgG; 1: 2000, Abcam, USA, ab150113) and goat anti-rabbit antibody to IgG (1:2000, Abcam, USA, ab150077) for 2 h. Finally, the protein expression was determined with an ECL kit. GAPDH was used as internal reference.

### Dual-Luciferase reporter assay

The wild type and mutant type 3′-UTR region of circ-STC2 and TFR2 luciferase reporter vectors were designed and synthesized by Guangzhou RiboBio Co., Ltd. The cells were tansfected with miR-486-3p mimic or mimic nc and the wild type or mutant of circ-STC2 or TFR2 for 48 h. Then cells were lysed to detect the luciferase activities using a Luciferase Reporter Assay Kit (BioVision Tech, USA). The firefly luciferase activity was normalized to Renilla luciferase activity.

### RNA pull-down assay

The NPCs were transfected with biotinylated miR-486-3p probe or biotinylated NC probe. After transfection for 48 h, the cells were incubated with cell lysate (Ambion, Austin, Texas, USA) for 10 min. The cleavage was incubated with beads precoated with M-280 streptavidin (Sigma) at 4 ℃ for 3 h. Then, the bound RNA was purified by Trizol (Boyetime), and the circ-STC2 or TFR2 expression was measured with RT-qPCR.

### Statistical analysis

Each experiment was perform three times. The data in this study were analyzed using SPSS 20.0 and presented as mean ± SD. Student’s *t* test was used in two groups, and two-way ANOVA was used among multiple groups for difference analysis. *P* < 0.05 was considered statistically significant.

## Results

### Circ-STC2 expressions were up-regulated in IDD

**Fig. 1:**
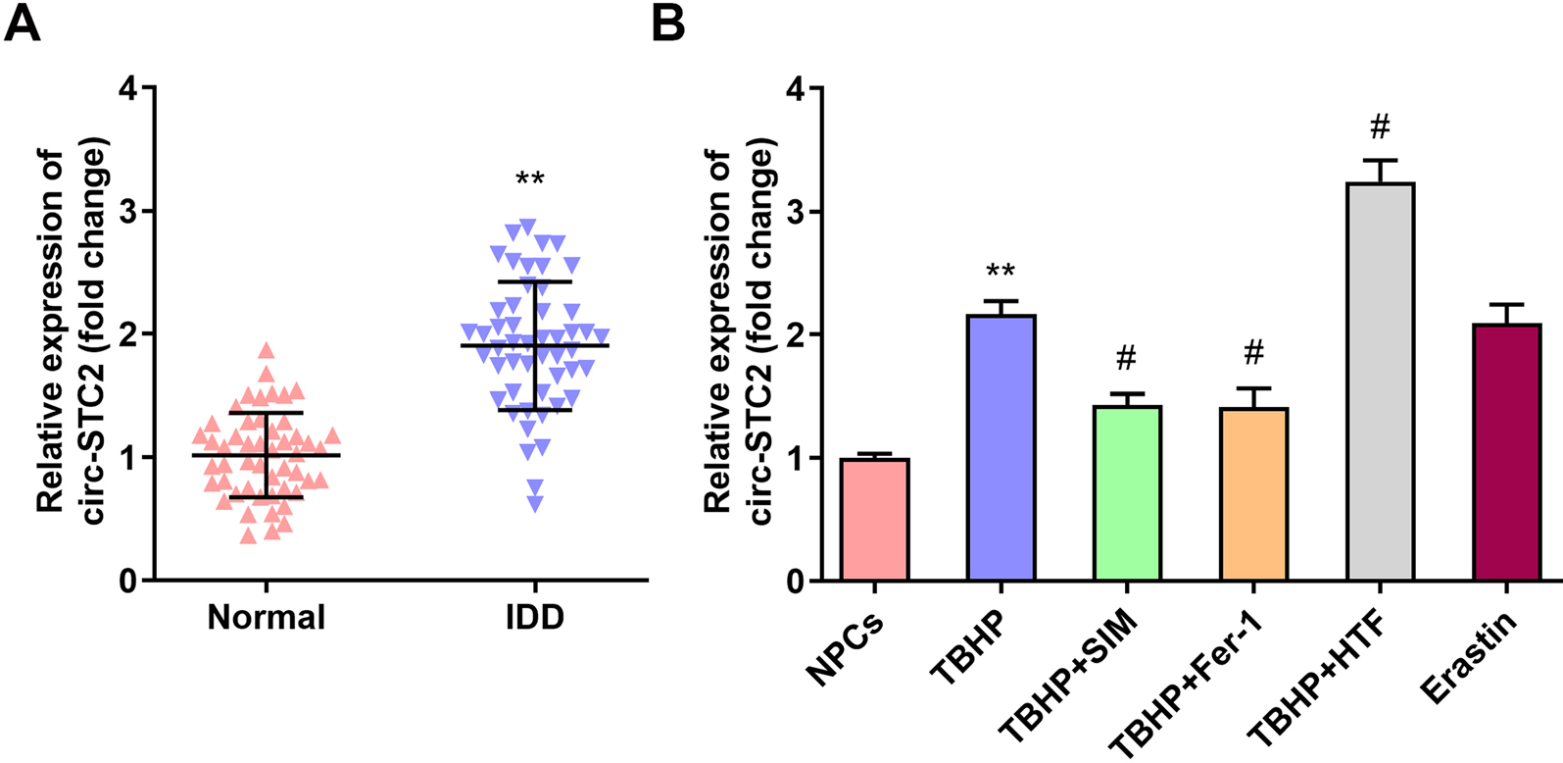
Circ-STC2 expressions were up-regulated in IDD. **A** The circ-STC2 expressions of IDD tissues were detected by RT-qPCR. **B** The circ-STC2 expressions of TBHP treated NPCs were detected by RT-qPCR after SIM, Fer-1, HTF and Erastin treatment. **P < 0.01 versus Nomal group or NPCs group; #*P* < 0.05 versus TBHP group

As shown in Fig. 1A, the circ-STC2 expressions were up-regulated in IDD. In addition, SIM was treated to cells as a positive control of Fer-1 to inhibit TBHP-induced injury. The HTF treatment was used to verify whether increasing the iron content would aggravate the TBHP-induced cell injury. Moreover, in this study, we also directly treated NPCs with Erastin to confirm ferroptosis could cause cell injury similar with TBHP treatment. The results showed that the circ-STC2 expression was up-regulated in TBHP treated NPCs, while SIM and Fer-1 treatment down-regulated the circ-STC2 expressions. Moreover, HTF treatment up-regulated the circ-STC2 expressions of TBHP treated NPCs (Fig. 1B).

### Knockdown of circ-STC2 promoted cell viability and inhibited ferroptosis of TBHP treated NPCs

**Fig. 2:**
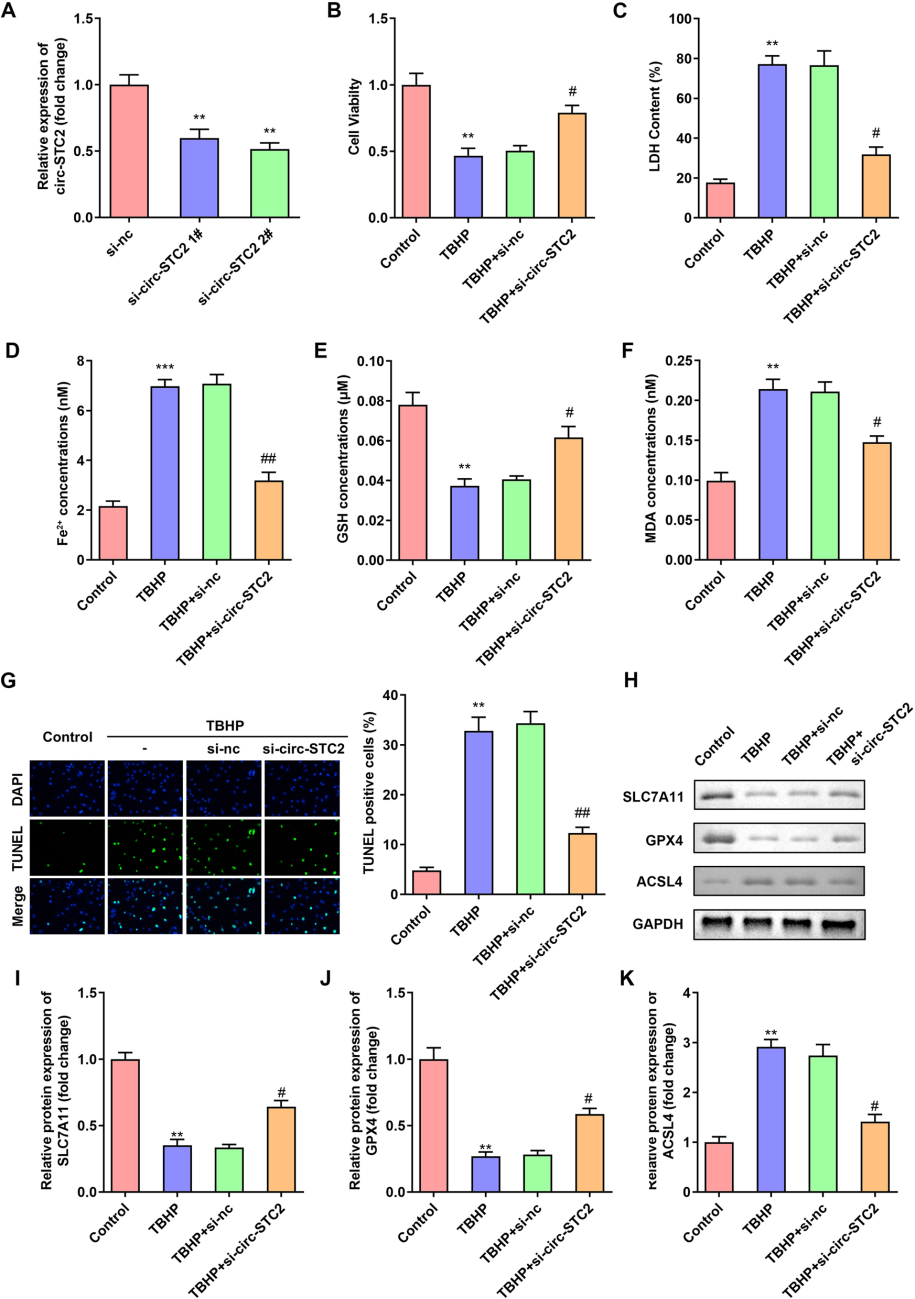
Knockdown of circ-STC2 promoted cell viability and inhibited ferroptosis of TBHP treated NPCs. **A** Validation of transfection efficiency of si-circ-STC2. NPCs were transfected with si-circ-STC2. **B** Cell viability was detected by CCK-8 assay. **C**–**F** The LDH, Fe^2+^, GSH and MDA levels of NPCs were measured with corresponding kits. **G** The cell death of NPCs was analyzed by TUNEL staining. **H**–**K** The protein expressions of SLC7A11, GPX4 and ACSL4 were detected bu western blot. **P < 0.01, ***P < 0.001 versus Control group; #*P* < 0.05, ##*P* < 0.01 versus TBHP + si-nc group

Next, after si-circ-STC2 1# and si-circ-STC2 2# transfection, the circ-STC2 expressions were significantly down-regulated in NPCs, which was more potent in si-circ-STC2 2# group (Fig. 2A). Therefore, si-circ-STC2 2# was used in further experiments. Si-circ-STC2 significantly alleviated inhibition of NPC cell viability induced by TBHP (Fig. 2B). In addition, circ-STC2 knockdown remarkably antagonized the effects of TBHP and decreased the LDH, Fe^2+^ and MDA levels, and increased the GSH levels (Fig. 2C–F). Furthermore, the TUNEL staining showed that TBHP-induced cell death was abated by circ-STC2 knockdown (Fig. 2G). What’s more, si-circ-STC2 increased the protein expressions of SLC7A11 and GPX4 and decreased ACSL4 (Fig. 2H–K).

### Circ-STC2 targeted miR-486-3p in TBHP treated NPCs

**Fig. 3:**
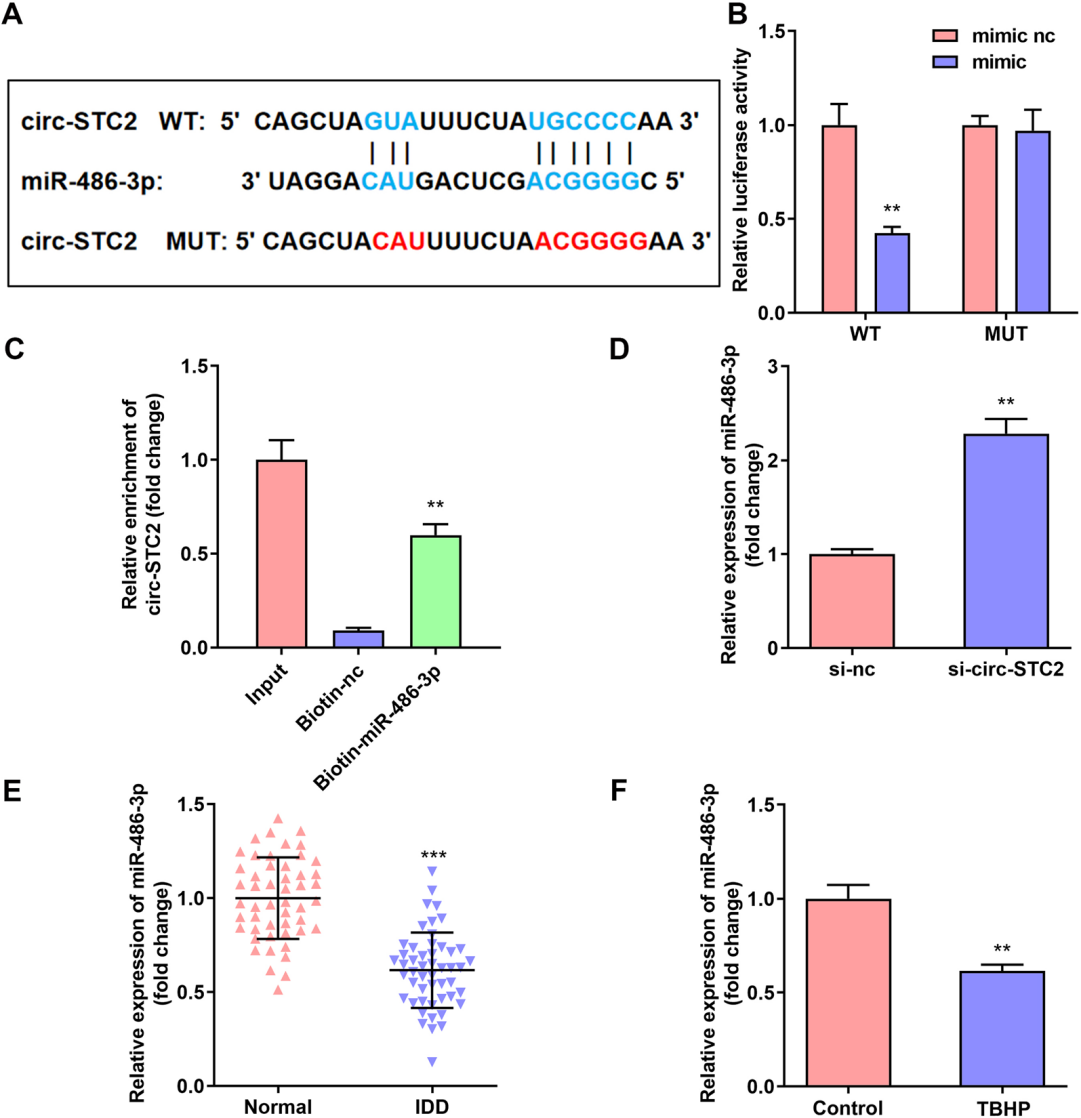
Circ-STC2 targeted miR-486-3p in TBHP treated NPCs. **A** Bioinformatics predicted the binding sites between circ-STC2 and miR-486-3p. Dual-luciferase reporter (**B**) and RNA pull-down assays **C** confirmed that miR-486-3p was a target of circ-STC2 in NPCs. **D** The miR-486-3p expressions in NPCs were detected by RT-qPCR after si-circ-STC2 transfection. The miR-486-3p expressions in IDD tissues (**E**) and TBHP treated NPCs **F** were detected by RT-qPCR. **P < 0.01

Starbase 3.0 (http://starbase.sysu.edu.cn/) was used to predict the target miRNA of circ-STC2. Figure 3A showed the binding sites between miR-486-3p and circ-STC2. The luciferase activity of the cells was significantly decreased after miR-486-3p mimic and WT-STC2 transfection (Fig. 3B). RNA pull-down assay further confirmed the interaction between miR-486-3p mimic and circ-STC2 (Fig. 3C). Furthermore, miR-486-3p expression was significantly up-regulated after si-circ-STC2 transfection (Fig. 3D). MiR-486-3p expression was significantly reduced in IDD tissues (Fig. 3E) and TBHP treated NPCs (Fig. 3F).

### Downregulated miR-486-3p reversed the effects of si-circ-STC2 on cell viability and ferroptosis of TBHP treated NPCs

**Fig. 4:**
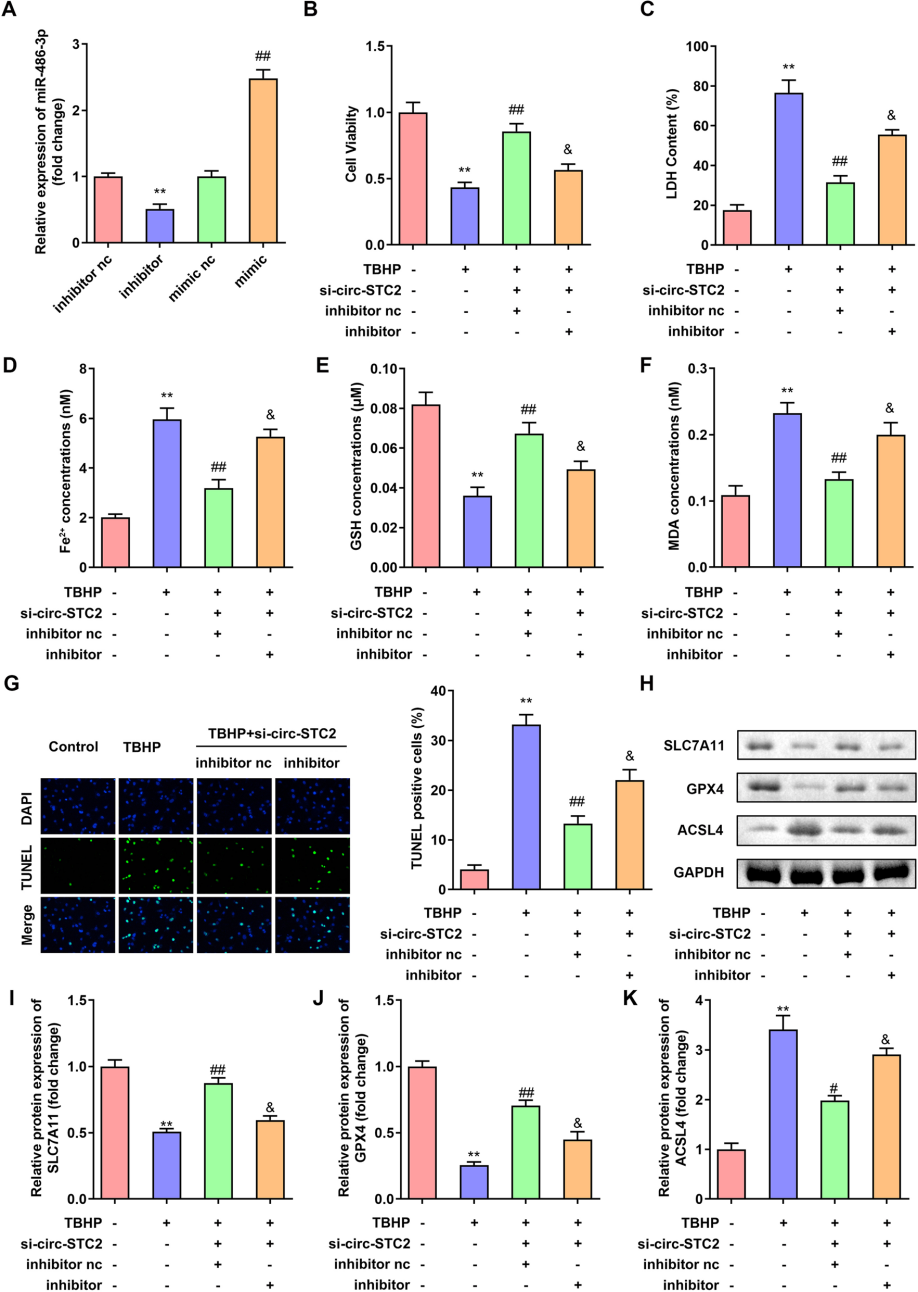
Knockdown of miR-486-3p reversed the effects of si-circ-STC2 on cell viability and ferroptosis of TBHP treated NPCs. **A** Validation of transfection efficiency of miR-486-3p mimic and inhibitor. NPCs were transfected with si-circ-STC2 and miR-486-3p inhibitor. **B** Cell viability was detected by CCK-8 assay. **C**–**F** The LDH, Fe^2+^, GSH and MDA levels of NPCs were measured with corresponding kits. **G** The cell death of NPCs was analyzed by TUNEL staining. **H** The protein expressions of SLC7A11, GPX4 and ACSL4 were detected bu western blot. **P < 0.01 versus Control group; ##*P* < 0.01 versus TBHP group; & *P* < 0.05 versus TBHP + si-circ-STC2 + inhibitor nc group

As shown in Fig. 4A, the miR-486-3p expression was detected by RT-qPCR assay. The results showed that the miR-486-3p expression was down-regulated after miR-486-3p inhibitor transfection, and up-regulated after miR-486-3p mimic transfection. Compared with the cells in the TBHP + si-circ-STC2 + inhibitor nc group, the cell viability was significantly decreased in the TBHP + si-circ-STC2 + inhibitor group (Fig. 4B). In addition, downregulated miR-486-3p significantly increased LDH, Fe and MDA levels and decreased GSH levels (Fig. 4C-F). Furthermore, downregulated miR-486-3p abrogated the effects of circ-STC2 knockdown and promoted cell death of NPCs (Fig. 4G). What’s more, miR-486-3p inhibitor reduced the protein expressions of SLC7A11 and GPX4 and increased ACSL4 protein expressions (Fig. 4H–K).

### TFR2 was a target gene of miR-486-3p

**Fig. 5:**
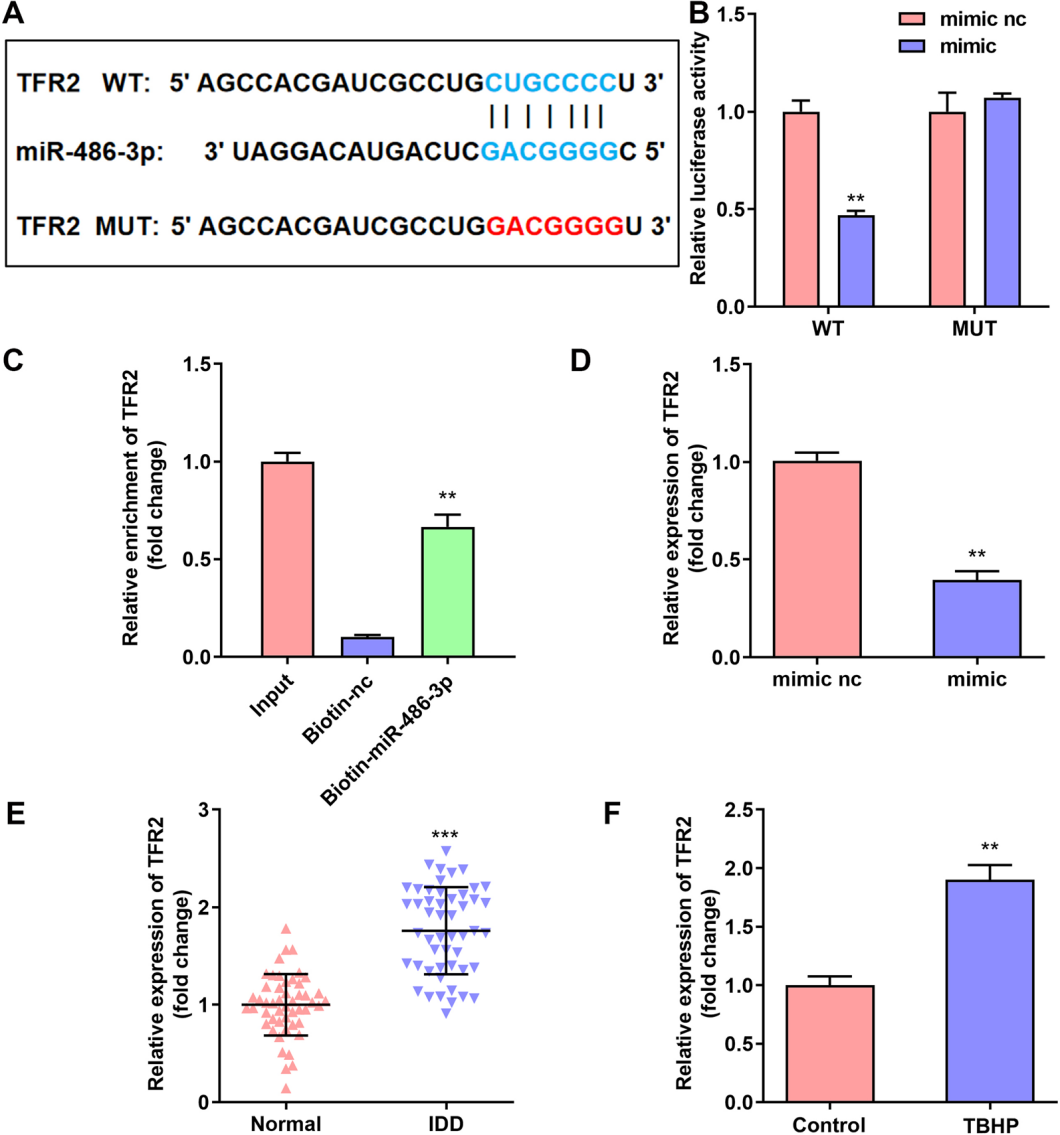
TFR2 was a target gene of miR-486-3p in TBHP treated NPCs. **A** Bioinformatics predicted the binding sites between miR-486-3p and TFR2. Dual-luciferase reporter (**B**) and RNA pull-down assays **C** confirmed that TFR2 was a target of miR-486-3p in NPCs. **D** The TFR2 expressions in NPCs were detected by RT-qPCR after miR-486-3p mimic transfection. The TFR2 expressions in IDD tissues (**E**) and TBHP-treated NPCs **F** were detected by RT-qPCR. **P < 0.01

TargetScan 7.2 (http://www.targetscan.org/) showed that TFR2 was a target gene of miR-486-3p (Fig. 5A). The luciferase activity was significantly decreased after co-transfection with miR-486-3p mimic and TFR2 3’UTR (Fig. 5B). In addition, the results of RNA pull-down assay demonstrated that compared with Biotin-nc group, the Biotin-miR-486-3p was significantly enriched in the TFR2 group (Fig. 5C). Furthermore, we found that TFR2 expression is down-regulated after miR-486-3p mimic transfection (Fig. 5D). And TFR2 expression is up-regulated in IDD tissues (Fig. 5E) and TBHP treated NPCs (Fig. 5F).

### Overexpression of TFR2 reversed the effects of miR-486-3p mimic on cell viability and ferroptosis of TBHP treated NPCs

**Fig. 6:**
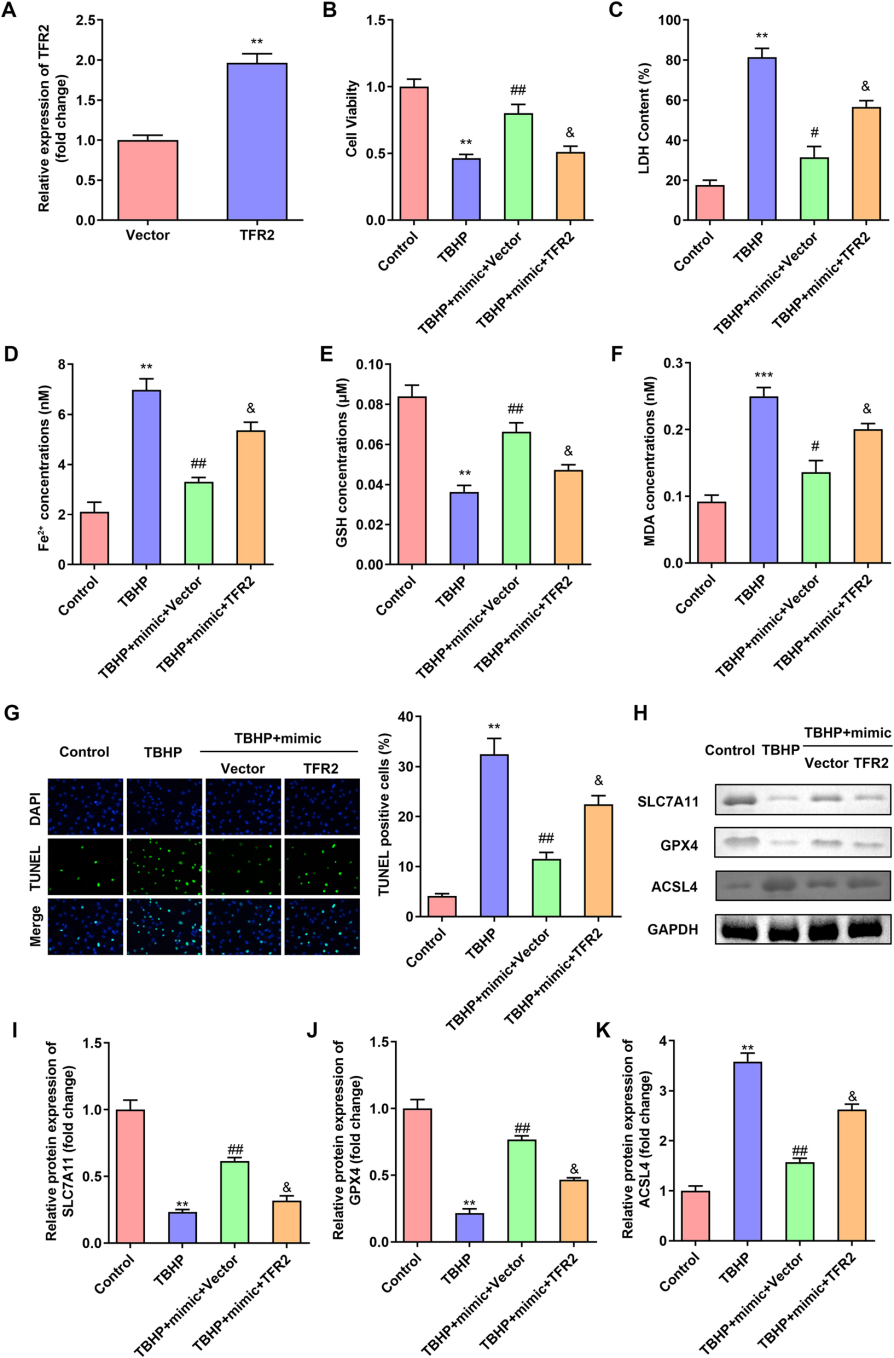
Overexpression of TFR2 reversed the effects of miR-486-3p mimic on cell viability and ferroptosis of TBHP treated NPCs. **A** Validation of transfection efficiency of TFR2. NPCs were transfected with miR-486-3p mimic and TFR2. **B** Cell viability was detected by CCK-8 assay. **C**–**F** The LDH, Fe^2+^, GSH and MDA levels of NPCs were measured with corresponding kits. **G** The cell death of NPCs was analyzed by TUNEL staining. **H**–**K** The protein expressions of SLC7A11, GPX4 and ACSL4 were detected by western blot. **P < 0.01, ***P < 0.001 versus Control group; #*P* < 0.05, ##*P* < 0.01 versus TBHP group; & *P* < 0.05 versus TBHP + miR-486-3p mimic + Vector group

As shown in Fig. 6A, the TFR2 expression was up-regulated after TFR2 transfection. Overexpressed TFR2 significantly suppressed cell viability (Fig. 6B). Upregulation of TFR2 significantly increased the LDH, Fe^2+^ and MDA levels and decreased GSH levels (Fig. 6C–F). Furthermore, TFR2 alleviated the effects of miR-486-3p mimic on NPC cell death and the expression of ferroptosis-related protein expression, including SLC7A11, GPX4, and ACSL4 (Fig. 6G–K). As shown in supplementary Fig. 1, we found that miR-486-3p mimic had no effects on the normal NPCs, while TRF2 showed the same effect as in TBHP stimulated NPCs.

## Discussion

In present study, circ-STC2 promoted the development of IDD. Knockdown of circ-STC2 promoted the cell viability and inhibited the ferroptosis of NPCs through regulating the miR-486-3p/TFR2 axis.

It has been reported that ferroptosis is related to the IDD pathogenesis [21, 22]. Hence, in this study, we found that after ferroptosis inhibitor treatment, the circ-STC2 expressions were significantly decreased. We suspected that the role of circ-STC2 in IDD development might related to ferroptosis. Solute carrier family 7 member 11 (SLC7A11) is one of the main protein that regulates the ferroptosis. Overexpression of SLC7A11 can significantly inhibit the ferroptosis [23]. In addition, ferroptosis is closely related to the production of lipid peroxide (MDA), also suppressed by the lipid repair system of glutathione (GSH) and glutathione peroxidase 4 (GPX4) [24]. Acyl coenzyme A synthetase long chain family member 4 (ACSL4) is a member of the ACSL family. It has been found that ACSL4 is a key gene in the ferroptosis pathway and becomes an essential molecule for ferroptosis by participating in the synthesis of membrane phospholipids [25]. In this study, the development of IDD was accompanied with the increase in concentration of Fe^2+^, LDH and MDA and decrease in GSH level. Furthermore, TBHP treatment significantly decreased the protein expression of SLC7A11 and GPX4, and increased the ACSL4 expressions. Yang et al. [21] demonstrate that inhibition of ferroptosis could relieve the IDD development, suggesting that inhibition of ferroptosis may be a promising therapy for IDD.

CircRNAs are involved in various physiological functions [26, 27]. Accumulating evidence showed that aberrant expressed circRNAs are associated with the initiation and development of IDD [19, 28]. In current study, circ-STC2 was up-regulated in IDD tissues and TBHP treated NPCs. circ-STC2 or TFR2 contains miR-486-3p target sites. Therefore, we speculated that the role of circ-STC2 are mediated by miR-486-3p/TFR2 axis. Recently, miRNAs exhibited the effective abilities on the prevention and treatment of IDD, specifically on regulating IDD-related cell processes, such as NPCs proliferation and apoptosis [29–31]. It has been reported that miR-486 is down-regulated in IDD tissues [32], which was similar to our results. Cui et al. [33] also demonstrated that miR-486-3p promoted the proliferation and ECM synthesis of NPCs in IDD. Thence, miR-486-3p may protect against IDD. In this study, downregulation of miR-486-3p alleviated the effects of circ-STC2 knockdown on the cell viability and ferroptosis of TBHP treated NPCs. These results suggesting that circ-STC2 exerted its roles in the development of IDD via sponging miR-486-3p.miRNAs participate in IDD development via negative regulating the target genes. For example, miR-182-5p activated mitophagy and inhibited apoptosis of TBHP stimulated NPCs via regulating the Sirtuin 1 expression [34]. miR-150-5p decreased the IL-6, TNF-α and IL-1β levels in NPCs via down-regulating the matrix metallo protease 16 expressions [35]. In current study, miR-486-3p directly targeted TFR2. Transferrin receptor (TFR) is a transmembrane glycoprotein whose function is to mediate iron absorption through interaction with transferrin. In normal cells, the level of receptor expression is relatively low [36]. Due to the increased demand for iron in rapidly growing tumor cells, the expression of the iron transfer protein receptor increased significantly in tumor cells [37]. TFR2 is mainly expressed in the liver, and its main function may be to regulate and maintain the dynamic balance of iron ions in the body [38]. However, the role of TFR2 in IDD has not been reported. This study confirmed that TFR2 was up-regulated in IDD tissues and overexpression of TFR2 could reversed the role of miR-486-3p mimic in TBHP treated NPCs. These results indicated that circ-STC2 participates in the IDD pathogenesis via miR-486-3p/TFR2 axis.

In conclusion, knockdown of circ-STC2 promoted the cell viability and inhibited the ferroptosis of via targeting miR-486-3p/TFR2 axis. This may provide a novel strategy for IDD. However, there were still some limitations in this study. Due to limitations in conditions, this study did not conduct animal experiments to further demonstrated the role of circ-STC2/miR-486-3p/TFR2 axis in IDD. In our future research, we will focus on this in vivo experiments and conduct a more comprehensive exploration.

## Supplementary Information

**Additional file1**. **Figure S1**. Effects of miR-486-3p mimic and TFR2 on cell viability and ferroptosis of NPCs. **A** Cell viability was detected by CCK-8 assay. **B**–**E** The LDH, Fe2+, GSH and MDA levels of NPCs were measured with corresponding kits. **F**–**G** The cell death of NPCs were analyzed by TUNEL staining. **H**–**K** The protein expressions of SLC7A11, GPX4 and ACSL4 were detected by western blot. *P<0.05, **P<0.01 versus mimic + Vector group.

## Data Availability

The datasets used and/or analyzed during the current study are available from the corresponding author on reasonable request.
