# Supplementary figures and images for: Circ-STC2 promotes the ferroptosis of nucleus pulposus cells via targeting miR-486-3p/TFR2 axis
Source: J Orthop Surg Res. 2023 Jul 21;18:518. doi: 10.1186/s13018-023-04010-1 (PMC10362726; doi:10.1186/s13018-023-04010-1)

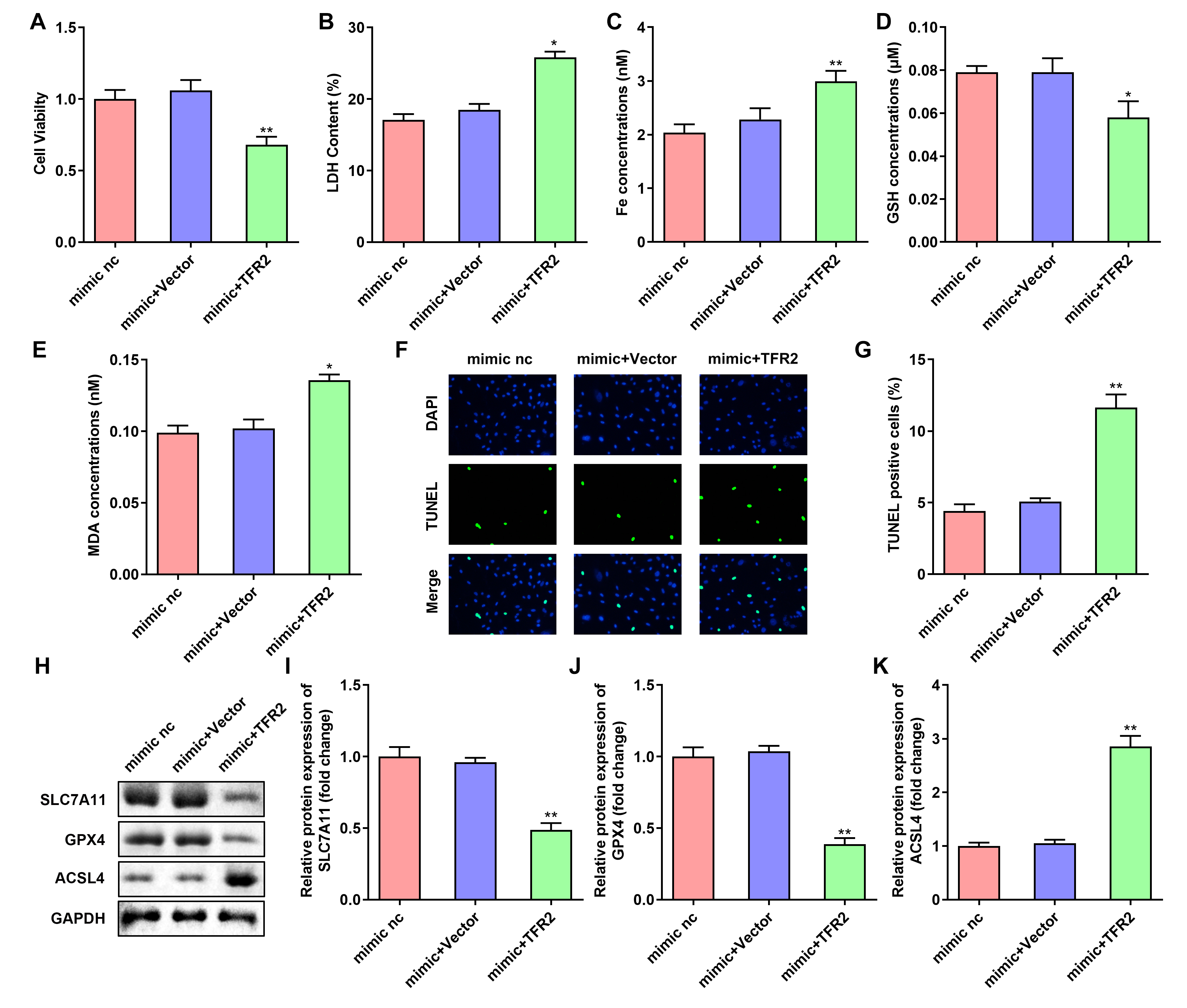

Supplement: Supplementary file 1 — Additional file1. Figure S1. Effects of miR-486-3p mimic and TFR2 on cell viability and ferroptosis of NPCs. A Cell viability was detected by CCK-8 assay. B–E The LDH, Fe2+, GSH and MDA levels of NPCs were measured with corresponding kits. F–G The cell death of NPCs were analyzed by TUNEL staining. H–K The protein expressions of SLC7A11, GPX4 and ACSL4 were detected by western blot. *P<0.05, **P<0.01 versus mimic + Vector group. [file 13018_2023_4010_MOESM1_ESM.jpg]
